# Quantitative Determination of Lercanidipine Enantiomers in Commercial Formulations by Capillary Electrophoresis

**DOI:** 10.1155/2015/294270

**Published:** 2015-03-02

**Authors:** Luciana Pereira Lourenço, Fernando Armani Aguiar, Anderson Rodrigo Moraes de Oliveira, Cristiane Masetto de Gaitani

**Affiliations:** ^1^Department of Pharmaceutical Sciences, Faculty of Pharmaceutical Sciences of Ribeirão Preto, University of São Paulo, 14040-903 Ribeirão Preto, SP, Brazil; ^2^Departament of Chemistry, Faculty of Philosophy, Sciences and Letters of Ribeirão Preto, University of São Paulo, 14040-901 Ribeirão Preto, SP, Brazil

## Abstract

An enantioselective method based on capillary electrophoresis (CE) using cyclodextrin (CD) as chiral selector was developed and validated for determination of lercanidipine (LER) enantiomers, a drug calcium channel blocker which exerts antihypertensive effects of long duration, in a pharmaceutical formulation. Optimum separation of LER enantiomers was obtained on a 50 cm × 50 *μ*m id capillary using a sodium acetate buffer solution 200 mmol/L pH 4.0 containing 10 mmol/L of 2,3,6-o-methyl-*β*-cyclodextrin (TM-*β*-CD) as background electrolyte. The capillary temperature and voltage were 15°C and 25 kV, respectively, hydrodynamic injection and detection at 237 nm. Linearity was obtained in the range 12.5–100 *μ*g/mL for both enantiomers (*r* ≥ 0.995). The RSD (%) and relative errors (*E*, %) obtained in precision and accuracy studies (intraday and interday) were lower than 5%. After validation, the method was applied to quantify the enantiomers of LER in commercial tablets and the results were satisfactory in terms of accuracy and precision, both less than 5%. Therefore, this method was found to be appropriate for enantioselective quality control of LER enantiomers in pharmaceutical formulations.

## 1. Introduction

**Figure 1:**
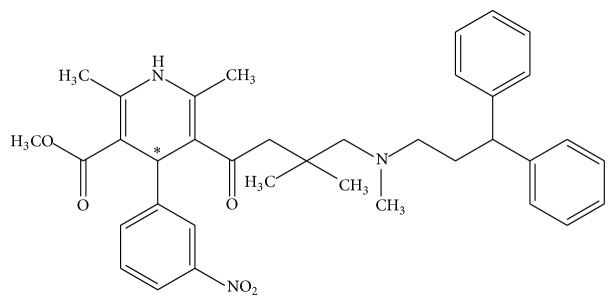
Chemical structure of LER (∗: chiral center).

Lercanidipine (LER), (±) 2-[(3,3-diphenylpropyl)methylamine]-1,1-dimethylethyl methyl 1,4-dihydro-2,6-dimethyl-4-(3-nitrophenyl)-3,5 pyridinedicarboxylic ester (Figure 1), is a dihydropyridine type calcium-channel blocker known to effectively and safely reduce high levels of blood pressure, with a low adverse effect [1, 2]. The calcium channel antagonists inhibit the influx of calcium ions through voltage-operated calcium channels located in the cell membrane [3–5].

The chemical structure of LER is characterized by the presence of a side chain containing a 3,3-diphenylpropyl-methylamine-2-methyl-2-propyl group that was introduced to improve the lipophilic properties and the activity duration of the drug. Owing to the presence of asymmetric ester moieties, LER has a chiral carbon atom in position 4 of the dihydropyridine ring (Figure 1) [6]. It is commercially available as a racemic mixture of (R)- and (S)-enantiomer. The pharmacological effects of the enantiomers of LER essentially resides in the (S)-enantiomer. Studies* in vitro* showed that (S)-enantiomer has about 100–200 times higher affinity for calcium channels than the (R)-enantiomer. Consequently, the pharmacological effects of LER are mainly related to the (S)-enantiomer [7–9].

Since the fact that many enantiomers of racemic drugs may exhibit differences in pharmacological effects, there is a need for the development of enantioselective methods [10–12].

To the occurrence enantioselective separation to chiral analytes, the use of chiral selectors is necessary. The main chiral selector is cyclodextrins (CD) for possessing several properties that make them attractive, such as low UV absorption, solubility and different polarity, and high degree of enantiomeric discrimination. The most likely mechanism for enantioseparation using CDs involves the inclusion (or partially inclusion) of the chiral analyte in the groove and the establishment of secondary interactions with hydroxyl groups on the rim of the CD. The difference in the constants of inclusion between the enantiomers and the CDs is responsible for enantioresolution since the diasteroisomers formed will have different mobilities [13].

There are several methods that describe the analysis of LER not only in pharmaceutical formulations but also in biological fluids. All these methods have been using high performance liquid chromatography (HPLC) for the analysis [2, 12, 14]. HPLC methods have been reported for the enantioselective analysis of LER employing different kind of stationary phase [15–18]. On the other hand, no method has been cited in the literature for the enantioselective analysis of LER by CE until now. CE is an effective technique for enantioseparations and presents advantages as high efficiency and versatility, rapid analysis, and low operating costs [19–22].

In this paper, a simple, rapid, and accurate CE method for the determination of LER enantiomers using cyclodextrins (CDs) as chiral selector was developed. CD derivatives were investigated as chiral selectors; the electrophoretic conditions were optimized and the method was validated and applied to quantify the LER enantiomers in commercial tablets.

## 2. Experimental

### 2.1. Chemicals and Reagents

LER, active pharmaceutical ingredient (API), was kindly supplied by Medley (São Paulo, Brazil) and commercial tablets were purchased from local commerce containing 10 mg of mixture racemic of LER per tablet (São Paulo, Brazil).

The stock solution (1000 *μ*g/mL) and working solutions containing racemic mixture (25, 50, 100, 150, and 200 *μ*g/mL) of LER were prepared in methanol. The solutions were stored frozen at −20°C and protected from direct light using amber glass material [17, 23]. Methanol, ethanol, and sodium acetate were obtained from Merck (Darmstadt, Germany). Sulfobutyl ether *β*-cyclodextrin (S-*β*-CD) and heptakis (2,3,6-tri-o-methyl)-*β*-cyclodextrin (TM-*β*-CD) were obtained from Fluka (Buchs, Switzerland). Hydroxypropyl *β*-cyclodextrin (HP-*β*-CD), 2-(N-morpholino)ethanesulfonic acid (MES), and diethylamine (DEA) were purchased from Sigma Aldrich (St. Louis, USA). Acetic acid was purchased from Labsynth (São Paulo, Brazil). Tris (2-amino-2-hydroxymethyl-propane-1,3-diol) and hexane were obtained from J. T. Baker (Phillipsburg, USA). Sodium hydroxide was purchased from Nuclear (São Paulo, Brazil) and hydrochloric acid was obtained from Zilquímica (São Paulo, Brazil). Water was purified using a Direct-Q 3 system from Millipore (Bedford, USA). All other chemicals employed were analytical or LC grade or other unless otherwise mentioned.

### 2.2. Apparatus and Analytical Conditions

Analyses were performed on a Beckman Coulter CE system (Fullerton, USA) P/ACE MDQ model consisting of an analyzer, an automatic sampler, and a diode array detector operating at 237 nm. 32 Karat Software for data acquisition was used. A fused-silica uncoated capillary obtained from Beckman Coulter (Fullerton, USA) of 50 *μ*m id, 60.2 cm total length, and 50.0 cm effective length was used in the analysis. Before the first use, the capillary was conditioned by rinsing with 1.0 mol/L NaOH for 30 min and then with water for 30 min at 25°C. At the beginning of each working day, the capillary was rinsed with 0.1 mol/L NaOH for 15 min and then with water for 15 min. The capillary was rinsed with 0.1 mol/L NaOH for 2.0 min, water for 2.0 min, and running buffer for 2.0 min between consecutive analyses. After daily use, the capillary was washed with 0.1 mol/L NaOH for 15 min and then with water for 15 min. The capillary was filled with water, when it was not in use.

### 2.3. Migration Order of the Enantiomers

The migration order of LER enantiomers was based on [17]. The migration order was established by analyzing the pure enantiomers of LER obtained by HPLC system from Shimadzu liquid chromatography (Kyoto, Japan) equipped with an LC-10 AS pump, an SCL-10 AVP system controller, and an SPD-10 UV–vis detector operating at 237 nm. It was accomplished using a Chiralpak AD column (250 × 4.6 mm, 10 *μ*m particle size, Chiral Technologies Inc. Exton, PA, USA) and mobile phase consisting of hexane : ethanol : diethylamine (97 : 3 : 0.3, v/v/v) at a flow rate of 1.0 mL/min [17]. The peaks of each enantiomer (S-LER and R-LER) were separately collected at the end of the detector; the solvent was evaporated and the residues were analyzed by CE under the conditions established in the present paper and the elution order established.

### 2.4. Validation of the Method for LER Determination

The method was validated for LER enantiomer analysis in standard solutions and tablet samples. Before starting the validation study, the conformity system was conducted. The system suitability test was also performed to evaluate the repeatability of the system. So, ten replicate injections of a standard solution containing 100 *μ*g/mL LER (racemic mixture) in the optimized analytical conditions were performed, and the migration time was evaluated.

The linearity of the method was determined by construction of calibration curve using five LER concentration levels (12.5, 25.0, 50.0, 75.0, and 100 *μ*g/mL) for each enantiomer. Three replicate injections of the standard solutions were made and the peak height of the electropherogram was plotted versus the concentrations of LER enantiomers to obtain the respective calibration graph. The data were then subjected to regression analysis by the least-square method to calculate the calibration equation and correlation coefficient (*r*).

The limit of detection (LOD) and limit of quantification (LOQ) values were calculated directly using the calibration curve. The LOD and LOQ were expressed as the ratio of the standard deviation for the slope of the calibration curve multiplied by 3 and 10, respectively, as defined by the International Conference on Harmonization (ICH) [24].

The precision and accuracy of the method were evaluated by within-day and between-day (two consecutive days) analysis in samples on three different concentrations, for each enantiomer (12.5, 50, and 100 *μ*g/mL). The results were expressed of relative standard deviation (RSD, %) for precision and relative error (*E*, %), respectively.

The robustness test was evaluated by 2-Level 2^4–2^ (sixteen experiments), performed by the selection of four factors into two levels (high and low), such as pH (4.0 ± 0.2), concentration of the sodium acetate buffer (200 mmol/L ± 5.0 mmol/L), TM-*β*-CD concentration (10 mmol/L ± 2.0 mmol/L), and voltage (25 kV ± 2.0 kV). Resolution and migration time of enantiomers of LER were the factors evaluated and processed by Minitab 15 statistical software (State College, PA, USA).

### 2.5. Sample Preparation of LER Tablets for Assay

Tablets with a declared content of 10 mg of racemic LER were analyzed. Ten tablets were accurately weighed then ground to a fine powder in a mortar and thoroughly mixed. An amount equivalent to 10 mg (declared) of racemic LER was weighed and transferred into a 10 mL volumetric flask and about 6 mL of methanol were added. The mixture was then sonicated for 10 min, allowed to rest for 10 min before bringing it up to volume. A 1.0 mL aliquot was then vested to another 10 mL volumetric flask, and then the volume was completed with methanol. This solution (nominal racemic concentration: 100 *μ*g/mL) was analyzed.

## 3. Results and Discussions

### 3.1. Method Optimization

#### 3.1.1. Determination of the Background Electrolyte (BGE)

The structure chemical of LER shows weakly basic characteristics [24], and an analytical strategy has been to employ acid buffer [25]. In addition, the choice of the buffer is a critic point of this analysis, because the electroosmotic flow (EOF) and electrophoretic mobility are sensitive to pH variation [26]. During the determination of BGE the CD was not used.

Several BGE were evaluated: phosphate sodium, Tris, MES, and sodium acetate. Initially the sodium phosphate buffer at a concentration of 100 mmol/L at pH 3.0 was tested, and no peaks were found until 30 min of analysis. After that, Tris buffer at concentration of 50 and 100 mmol/L at pH 4.0 and MES buffer at concentration of 50–200 mmol/L at pH 4.0 were evaluated, and no peak was found, too. So, the sodium acetate buffer at concentration of 100 mmol/L at pH 4.0 was evaluated, and one peak was observed corresponding the LER (racemic mixture). This explanation is due to the better interaction between the drug and ions of acetate buffer. Thus, the sodium acetate buffer was then chosen for further analyses.

#### 3.1.2. Type and Concentration of CD(s)

CD is most commonly used chiral selectors in enantioselective analyzes by CE. During the method optimization, the selection of the appropriate CD for chiral separation was performed considering physicochemical characteristics, the shape, and the size of the LER and the CD. So, the S-*β*-CD (negative CD derivative) and HP-*β*-CD (neutral CD derivative) were evaluated as chiral selectors. However, these CDs were not able to separate the enantiomers. On the other hand, baseline enantioseparations were achieved when TM-*β*-CD (neutral CD derivative) was used as chiral selector, probably due to a better CD inclusion analyte and/or a better interaction between LER and methyl groups present on this CD, which lead to difference in the constant inclusion and hence in a difference in the mobility of each enantiomer. Thus, TM-*β*-CD was chosen for the following experiments.

**Figure 2:**
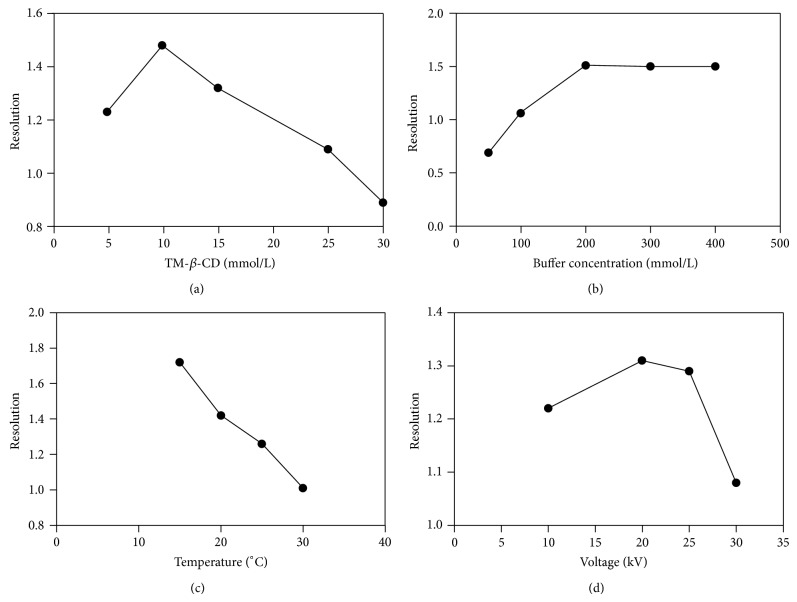
Effect of (a) TM-*β*-CD concentration (mmol/L); (b) buffer concentration (mmol/L); (c) temperature (°C) and (d) voltage (kV) on the resolution of LER enantiomers. Experimental conditions: fused-silica uncoated capillary 50 *μ*m id, 60.2 cm in total length and 50.0 cm in effective length, sodium acetate buffer pH 4.0, and 15°C, 25 kV, 237 nm, and injection: 10 seconds (at a pressure of 0.5 psi). Other conditions were changed according to each experiment.

**Figure 3:**
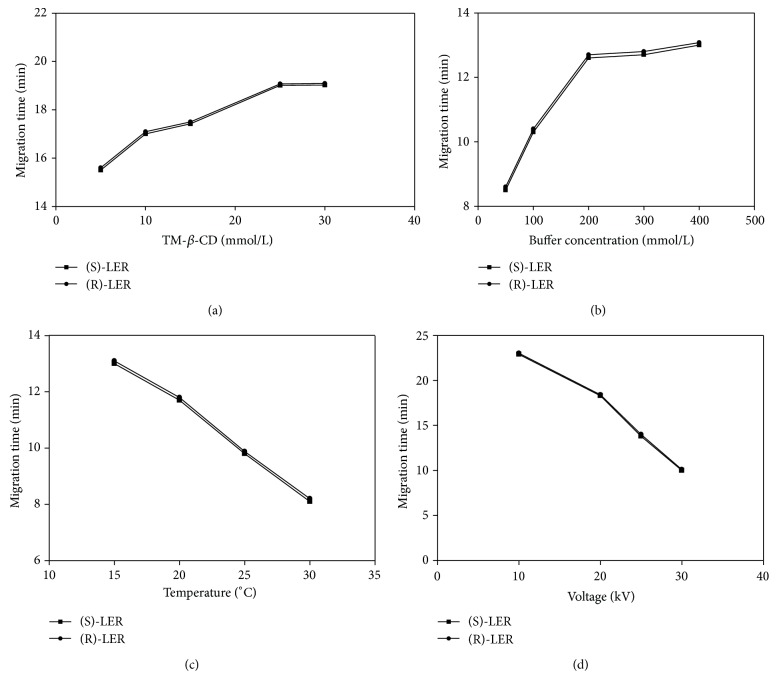
Effect of (a) TM-*β*-CD concentration (mmol/L); (b) buffer concentration (mmol/L); (c) temperature (°C) and (d) voltage (kV) on the migration time (min) of LER enantiomers. Experimental conditions: fused-silica uncoated capillary 50 *μ*m id, 60.2 cm in total length and 50.0 cm in effective length, sodium acetate buffer pH 4.0, and 15°C, 25 kV, 237 nm, and injection: 10 seconds (at a pressure of 0.5 psi). Other conditions were changed according to each experiment.

The CD concentration is important to achieve optimal experimental condition for separation. As can be seen in Figure 2(a), the resolution increased with increasing CD concentration until a level maximum. After this maximum of concentration point, a decreasing of resolution was observed. This is explained because the separation can be affected by the decrease in constant inclusion, which depends on the concentration of CD [21]. Since the migration time (Figure 3(a)) also increases with increasing CD concentration, due to mass increased of the complex formed [27], so the concentration selected of CD was 10 mmol/L.

#### 3.1.3. BGE: Concentration, pH, and Electrolysis

The concentration of the electrolyte solution should be evaluated once ionic strength can influence the internal heating of the capillary (Joule) and change the electrophoretic mobility [28]. In order to investigate the influence of the buffer concentration, its concentration was varied in the range of 50 to 400 mmol/L. According to Figure 2(b), the gradual increase in BGE concentration leads to an increase in the resolution. In addition, there was also an increase in the migration time (Figure 3(b)), which can be explained by the increased viscosity, which provides a reduction in the mobility of analytes, as well as the complex formed (analyte-CD) [29, 30]. Besides, the increase in the concentration of BGE provides an increase in the ionic strength of the medium and therefore decreasing the gradient in potential (zeta potential) in the wall of the capillary decreasing the EOF, which can have caused delay on the mobility of analytes [25, 26]. The best resolution with lower migration time was obtained using a sodium acetate buffer 200 mmol/L. Under these conditions, the current generated in the capillary was 80 *μ*A. At concentrations that exceeded 200 mmol/L, high electrical current resulting in peak broadening was observed. The optimal condition was achieved with 200 mmol/L sodium acetate buffer solution.

The correct choice of the BGE pH must be made carefully in order to achieve good resolution of the enantiomers. It can influence the EOF and the migration time, even as determining the charge of the solute and influence on chiral separation [31]. The most commonly condition used to separate basic compounds, such as LER, is to use low pH value, where the analyte is positively charged and migrates towards the cathode. Under these conditions, the EOF will be lower and the analyte will migrate due to their own electrophoretic mobility [29]. These factors allow more time for interaction between enantiomers and CDs. Thus, tests were made varying the pH from 3.0 to 5.0 using 10 mmol/L of TM-*β*-CD as a chiral selector and 200 mmol/L sodium acetate buffer. As can be seen in Table 1, the increase in BGE pH from 3.0 to 4.0 caused a slight increase in migration time and a significant increase in the plate number and resolution. On the other hand, the increase in pH from 4.0 to 5.0 caused a decrease in the plate number probably due to decreased in the ionization degree of the analyte at this pH value. Furthermore, at this pH there was an increase in migration time. As consequence of the results, pH 4.0 was selected as optimum value for the other experiments.

Finally, after selecting the buffer pH and concentration, the electrolysis test was carried out in order to evaluate the ability of the buffer to control the pH during the runs [25, 26, 32]. So, in the electrolysis test ten runs were made under the same analytical conditions. After that, the RSD (%) of retention time was <2.0% (Table 2). This way, the data obtained in these experiments prove that the BGE is stable up to ten runs.

#### 3.1.4. Temperature and Voltage

The temperature of the capillary is an important parameter to be controlled, because it proportionally affects the mobility and the kinetic inclusion of the analyte-CD [33]. Thus, the effect of temperature in the range from 15 to 30°C was investigated. During the experiments, an increase in the temperature decreased the enantiomeric separation and significantly reduced the migration time (Figures 2(c) and 3(c)). These facts are because of the changes in the constant inclusion and selectivity separation [34, 35] and the reduction in BGE viscosity of the medium, respectively. So, the temperature of 15°C was selected for further analyses.

The influence of the applied voltage over the resolution and migration times was studied in the range from +10 to +30 kV. According to Figure 2(d), the high voltages reduced resolution, probably due to excessive increase in the internal capillary temperature (Joule effect) and inefficiency in heat dissipation [35].  Figure 3(d) shows the significant reduction of migration time of the enantiomers with the gradual increase in voltage, since the increase in temperature provided a decrease in viscosity of the medium and, consequently, increased mobility of the analytes. Then, the voltage at +25 kV was chosen for the following analyses. At this condition the generated current was 52 *μ*A.

Thus, after optimization of all parameters, the best conditions for enantioseparation of LER were presented in Table 3.

#### 3.1.5. Determination of the Migration Order

**Figure 4:**
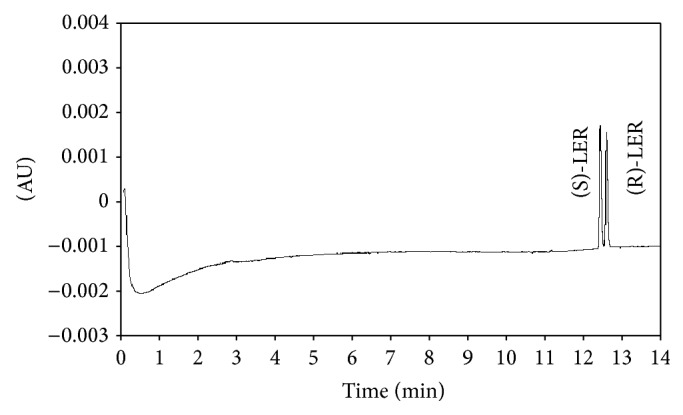
Electropherogram of migration order of LER enantiomers. Electrophoretic conditions: fused silica capillary of 50 *μ*m (id), 50 cm effective length, and sodium acetate buffer 200 mmol/L, pH 4.0, TM-*β*-CD: 10 mmol/L, 15°C, 25 kV.

The migration order was determined as described in material and methods section. The peaks were collected from the HPLC and separately injected in CE on the conditions determined by this study. The results showed that the first peak collected was (S)-LER, while the second peak collected was (R)-LER (Figure 4). The analyses were based in the enantiomers migrated on the electropherogram.

### 3.2. Method Validation

A system suitability test of the electrophoretic system was performed before validation. Fivefold injections of standard solutions were made (100 *μ*g/mL) and the migration time, asymmetry, plate number, and resolution were determined. The results were satisfactory according to the recommendation of USP [36] (Table 4). At this context, these parameters showed that the equipment was able for analyses.

Linear regression analyses were performed by plotting the ratio of peak height (*y*) versus theoretical enantiomer concentrations (*x*). The method proved to be linear over the concentration range from 12.5 to 100 *μ*g/mL for each enantiomer of LER, with correlation coefficient *r* > 0.995. The LOD and LOQ were calculated using the parameters of the analytical curve (Table 5).

In order to evaluate the precision and accuracy of the CE method, these were evaluated by calculating RSD (%) and *E* (%), respectively. Three determinations of LER enantiomers at three different levels of concentrations (low, middle, and high) in two consecutive days under the same experimental conditions were made (Table 5). The values of accuracy (%) and RSD (%) were less than 5% for the peak height in both experiments (within-day and between-day) for the each enantiomer. This performance suggests that the method is completely suitable for the quantitative determination of the studied drug.

Robustness, which is the measure of the method's capacity to remain unaffected by small but deliberate changes in electrophoretic conditions, was studied for LER standard solutions by analyzing them under conditions slightly changed from the defined method. Voltage, CD concentration, buffer concentration, and pH buffer were statistically significant (*α* = 0.05) at the levels evaluated for migration time and resolution. Therefore, these parameters should be controlled during analysis.

### 3.3. Application of the Method to the Analysis of Tablets Containing LER

**Figure 5:**
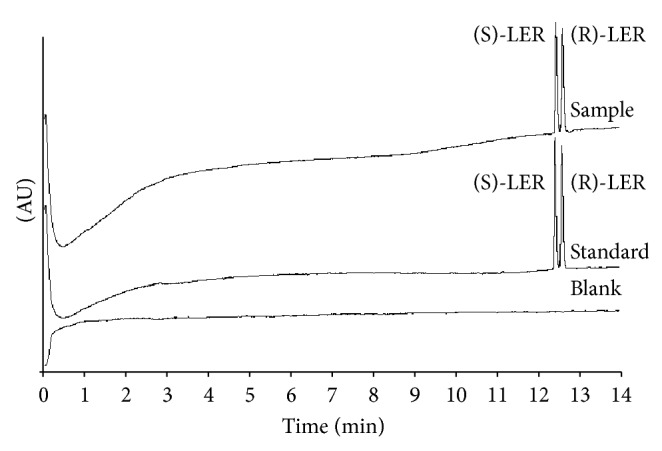
Typical electropherograms obtained following the analysis of Blank; a standard solution containing racemic LER and commercial tablets. Experimental conditions: fused-silica uncoated capillary 50 *μ*m id and 50.0 cm effective length, +25 kV, 200 mmol/L sodium acetate buffer pH 4.0 (containing 10 mmol/L of TM-*β*-CD), and 15°C, 237 nm; injection: 10 seconds (at a pressure of 0.5 psi).

The applicability of the proposed method was also evaluated by determination of LER enantiomers in commercially available tablets. The results obtained were satisfactory in terms of accuracy by the excellent recovery (% found) and precision (RSD, %) values (Table 6), according to British Pharmacopoeia 2004 recommendation [37]. The pharmaceutical formulation excipients of LER tablets did not interfere with the assay (Figure 5). The LER tablets (10 mg) analyzed are consistent with their specifications.

## 4. Conclusion

In summary, the enantiomers were quantified in the LER commercial tablets and the results showed that the formulation analyzed is within the specifications requested, with accuracy and precision of less than 5%. This method proved to be simple, selective, linear, precise, and accurate; besides it is also inexpensive and does not use polluting and toxic organic solvents. In addition, the method has the advantage of allowing for the baseline separation of enantiomers in less than 14 min with high efficiency. So, this study describes for the first time a validated method for the determining the enantiomers of LER by CE and can be routinely applied in the pharmaceutical industry as valuable alternative to chiral analyses that employ HPLC.

## Figures and Tables

**Table 1:** Results of the pH test for LER enantiomers analysis.

pH	Migration time (min)		Plates number		Resolution
	(S)-LER	(R)-LER	(S)-LER	(R)-LER	
3.0	11.44	11.57	83 362	75 509	0.79
4.0	11.66	11.82	215 394	190 436	1.31
5.0	17.33	17.52	189 374	153 614	1.15

**Table 2:** Electrolysis test of BGE for LER enantiomers analysis.

	S-(LER)	R-(LER)
Migration time (min)^ a^	13.6	13.8
RSD (%)^b^	0.6	0.6

^a^number of determination (*n*) = 5, ^b^RSD (%): relative standard deviation expressed as a percentage.

**Table 3:** Results of parameters optimization for LER enantiomers analyses.

Parameters	Optimization
Capillary	Fused-silica 50 *μ*m id 50.0 cm effective length 60.2 cm total length

BGE	Sodium acetate 200 mmol/L pH 4.0

CD	TM-*β*-CD 10 mmol/L

Temperature	15°C

Voltage	+25 kV

Detector	UV-DAD 237 nm

Injection	Hydrodynamic 0.5 psi 10 seconds

**Table 4:** Results of the system suitability test for LER enantiomer analysis.

Parameter	USP recommendation	Day	(S-LER)	(R-LER)
Migration time	RSD^a^ (%) <2	1	0.07	0.07
		2	0.3	0.3

Plates number	Values >2000	1	357 475	328 250
		2	354 674	324 078

Asymmetry	Values <2	1	1.2	1.2
		2	1.2	1.2

Resolution	Values >2	1	2.0	
		2	2.0	

Purity peak	Values^≈^ 1	1	0.9	0.9

^a^Values from five replicates analyses, RSD (%), relative standard deviation expressed as a percentage, and ^≈^values almost equal to.

**Table 5:** Linearity, LOD and LOQ, precision, and accuracy of the method for LER enantiomer analysis.

Limits	(S)-LER			(R)-LER		
LOD (*μ*g/mL)	0.8			0.6		
LOQ (*μ*g/mL)	2.4			1.9		

Slope (RSD)	5.8			1.0		

Intercept (RSD)	12.4			30.5		

Linearity						

Linear equation	*y* = 63.373*x* + 77.021			*y* = 56.208*x* + 141.89		
Correlation coefficient (*r*)	0,998			0,995		

Precision and accuracy						

Nominal concentration (*μ*g/mL)	12.5	50	100	12.5	50	100
Within-day (*n* ^a^ = 3)						
Analyzed concentration (*μ*g/mL)	12.4	49.8	101.1	12.4	51.2	100.1
Precision (RSD, %)^b^	3.4	4.4	3.1	4.5	3.7	3.2
Accuracy (*E*, %)^c^	−0.8	−0.4	1.1	−0.8	2.4	0.1
Between-day (*n* ^d^ = 2)						
Analyzed concentration (*μ*g/mL)	12.3	51.7	98.6	12.3	52.1	98.4
Precision (RSD, %)^b^	3.6	4.3	4.0	4.3	4.0	3.8
Accuracy (*E*, %)^c^	−1.6	3.4	−1.4	−1.6	4.2	−1.6

Calibration graphs were prepared in triplicate (*n* = 3) for concentrations of 12.5, 25, 50, 75, and 100 *μ*g/mL; *y* = *Ax* + *B*, where *y* is the peak height of LER enantiomers, *A* is the slope, *B* is the intercept, and *x* is the concentration of the measured solution in *μ*g/mL; ^a^ *n*: number of determinations; ^b^RSD (%): relative standard deviation expressed as percentage; ^c^ *E* (%): relative error expressed as percentage; ^d^ *n*: number of days.

**Table 6:** Determination of LER enantiomers in samples of commercial tablets.

Parameters	(S)-LER	(R)-LER
Quantity declared (mg)	5	5
Quantity found^a^ (mg)	5.0	5.0
RSD (%)	3.3	3.0
Recovery (%)	101.0	101.3

^a^Mean of five replicate analyses; RSD (%): relative standard deviation expressed as a percentage.
